# Supplementary material for: AnnotationBustR: an R package to extract subsequences from GenBank annotations
Source: PeerJ. 2018 Jul 3;6:e5179. doi: 10.7717/peerj.5179 (PMC6034590; doi:10.7717/peerj.5179)
Supplement: Supplemental Information 1 [file peerj-06-5179-s001.zip › SUPPLEMENT/README.docx]

**Borstein, S.R. & O’Meara, B. C. *AnnotationBustR*: An R package to extract subsequences from GenBank annotations. Submitted to *PeerJ*.**

**Authors:**

*****Samuel R. Borstein

University of Tennessee

Department of Ecology and Evolutionary Biology

569 Dabney Hall

University of Tennessee

Knoxville, TN, 37996

[sborstei@vol.utk.edu](mailto:sborstei@vol.utk.edu)

Brian O’Meara

University of Tennessee

Department of Ecology and Evolutionary Biology

569 Dabney Hall

University of Tennessee

Knoxville, TN, 37996

[bomeara@utk.edu](mailto:bomeara@utk.edu)

**Source code for AnnotationBustR is available through:**

**GitHub:** <https://github.com/sborstein/AnnotationBustR>

**CRAN**: <https://cran.r-project.org/package=AnnotationBustR>

**File List (files found within AnnotationBustR_Supplements.zip)**

AB_alginment.phy

AB_partitions.txt

Accessions.csv

AnnotationBustR_PerformanceTest.R

AnnotationBustR_PerformanceTest.RData

cp_taxa.txt

cpTimes.csv

GB_alginment.phy

GB_partitions.txt

mt_taxa.txt

mtTimes.csv

PHLAWD

RAxML_bipartitions_AB_Extract.tre

RAxML_bipartitions_GB_Extract.tre

rDNA_taxa.txt

rDNATimes.csv

TreeCompare.R

**Description**

AB_alginment.phy: Concatenated phylip formatted alignment of sixty-one species of minnows and five genes extracted using *AnnotationBustR* used to construct a phylogeny in RAxML.

AB_partitions.txt: Partitions used in RAxML to build phylogeny using the alignment of sequences extracted using *AnnotationBustR* in AB_alginment.phy

Accessions.csv: Accession table for sequences used in the comparison of trees constructed from *AnnotationBustR* extracted sequences and single gene sequences from GenBank. The column Mitogenome contains mitogenome accessions while those named for a single locus (i.e. nd2) are individual GenBank sequences.

AnnotationBustR_PerformanceTest.R: This is the code used for running the timing tests and making Figure 2 in the manuscript.

AnnotationBustR_PerformanceTest.RData: This is the associated RData file from running the timing tests in the AnnotationBustR_PerformanceTest.R file.

cp_taxa.txt: A file containing accession numbers for chloroplast genomes, of which 100 were randomly selected in AnnotationBustR_PerformanceTest.R for performance timings.

cpTimes.csv: A file containing the timing results from extracting 1-13 chloroplast coding sequences from 100 randomly selected chloroplast genomes.

GB_alginment.phy: Concatenated phylip formatted alignment of sixty-one species of minnows and five genes from individual GenBank sequences used to construct a phylogeny in RAxML.

GB_partitions.txt: Partitions used in RAxML to build phylogeny using the alignment of single GenBank sequences in GB_alginment.phy.

mt_taxa.txt: A file containing accession numbers for metazoan mitogenomes, of which 100 were randomly selected in AnnotationBustR_PerformanceTest.R for performance timings.

mtTimes.csv: A file containing the timing results from extracting 1-13 mitochondrial coding sequences from 100 randomly selected mitogenomes.

PHLAWD: Configuration files, taxon list, and keep files used for performing PHLAWD analyses.

RAxML_bipartitions_AB_Extract.tre: Bootstrapped phylogenetic tree of sixty leuciscine minnows and one outgroup for five mitochondrial gene sequences extracted using AnnotationBustR. Corresponds to the phylogeney in figure 3.

RAxML_bipartitions_AB_Extract.tre: Bootstrapped phylogenetic tree of sixty leuciscine minnows and one outgroup for five mitochondrial gene sequences obtained as single sequences from GenBank. Corresponds to the phylogeney in figure 3.

rDNA_taxa.txt: A file containing accession numbers for metazoan rDNA sequences, of which 100 were randomly selected in AnnotationBustR_PerformanceTest.R for performance timings.

rDNATimes.csv: A file containing the timing results from extracting 1-5 rDNA subsequences (18S rRNA, ITS1, 5.8S rRNA, ITS2, 28S rRNA) from 100 randomly selected metazoan rDNA sequences.

TreeCompare.R: This is the code used for comparing alignments and trees between *AnnotationBustR* and GenBank datasets.
